# Interpretable Differential Diagnosis of Non-COVID Viral Pneumonia, Lung Opacity and COVID-19 Using Tuned Transfer Learning and Explainable AI

**DOI:** 10.3390/healthcare11030410

**Published:** 2023-01-31

**Authors:** Md. Nazmul Islam, Md. Golam Rabiul Alam, Tasnim Sakib Apon, Md. Zia Uddin, Nasser Allheeib, Alaa Menshawi, Mohammad Mehedi Hassan

**Affiliations:** 1 Department of Computer Science and Engineering, BRAC University, Dhaka 1212, Bangladesh; 2 Software and Service Innovation, SINTEF Digital, 0373 Oslo, Norway; 3 Information Systems Department, College of Computer and Information Sciences, King Saud University, Riyadh 11543, Saudi Arabia

**Keywords:** COVID-19, transfer learning, explainable AI, X-ray imaging, lung opacity, non-COVID viral pneumonia

## Abstract

The coronavirus epidemic has spread to virtually every country on the globe, inflicting enormous health, financial, and emotional devastation, as well as the collapse of healthcare systems in some countries. Any automated COVID detection system that allows for fast detection of the COVID-19 infection might be highly beneficial to the healthcare service and people around the world. Molecular or antigen testing along with radiology X-ray imaging is now utilized in clinics to diagnose COVID-19. Nonetheless, due to a spike in coronavirus and hospital doctors’ overwhelming workload, developing an AI-based auto-COVID detection system with high accuracy has become imperative. On X-ray images, the diagnosis of COVID-19, non-COVID-19 non-COVID viral pneumonia, and other lung opacity can be challenging. This research utilized artificial intelligence (AI) to deliver high-accuracy automated COVID-19 detection from normal chest X-ray images. Further, this study extended to differentiate COVID-19 from normal, lung opacity and non-COVID viral pneumonia images. We have employed three distinct pre-trained models that are Xception, VGG19, and ResNet50 on a benchmark dataset of 21,165 X-ray images. Initially, we formulated the COVID-19 detection problem as a binary classification problem to classify COVID-19 from normal X-ray images and gained 97.5%, 97.5%, and 93.3% accuracy for Xception, VGG19, and ResNet50 respectively. Later we focused on developing an efficient model for multi-class classification and gained an accuracy of 75% for ResNet50, 92% for VGG19, and finally 93% for Xception. Although Xception and VGG19’s performances were identical, Xception proved to be more efficient with its higher precision, recall, and f-1 scores. Finally, we have employed Explainable AI on each of our utilized model which adds interpretability to our study. Furthermore, we have conducted a comprehensive comparison of the model’s explanations and the study revealed that Xception is more precise in indicating the actual features that are responsible for a model’s predictions.This addition of explainable AI will benefit the medical professionals greatly as they will get to visualize how a model makes its prediction and won’t have to trust our developed machine-learning models blindly.

## 1. Introduction

The novel coronavirus, taxonomically known as Severe acute respiratory syndrome coronavirus 2 or SARS-CoV-2 and labeled by the World Health Organization (WHO) as COVID-19, appeared in Wuhan, Hubei Province, China, before the end of 2019 [1] and has sparked unprecedented global fear. The World Health Organization declared this a public health emergency of worldwide concern and dubbed it a global pandemic due to the virus’s fast spread from human to human [2]. Thus, COVID-19 has proliferated globally and is practically dispersed everywhere. Overall, 664,581,427 cases of COVID-19, including 6,696,262 deaths, have been declared by the WHO since 05:05 pm central European summer time (CEST) on 30 December 2022 [3]. Coronaviruses (CoVs) cause respiratory and intestinal diseases in human and animal populations [4]. The period of incubation (i.e., from commencement of symptoms) is between 2 and 14 days, a median of 4 and 5 days. Most persons (i.e., about 80%) who become infected do not have symptoms or mild illnesses. As a result, people who are infected with the virus but are unaware of it can infect others, allowing the infection to spread widely. In situations of age, smoking, or other major medical diseases such as cancer, heart, lungs, kidney, or liver illness, diabetes, immunocompromising conditions, Sickle cell disease, or obesity, the risk of serious disease and death increases with COVID-19 [5,6]. Additionally, variants of COVID such as SARS-COV-2, Middle East respiratory syndrome (MERS) are spreading faster and having a negative impact on life and the economy, something humans have never experienced in recent history [7].

Effective screening of affected patients is crucial in the fight against COVID-19, as it enables those infected to receive prompt treatment and care, as well as isolation to prevent the virus from spreading [8]. The most common clinical testing approach in COVID-19 patients using respiratory specimens for the purpose of testing is reverse transcription polymerase chain reaction (RT-PCR), which is expensive, less-sensitive and requires specialized medical personnel [9]. Moreover, there are various types of inexpensive antigen tests, among which BinaxNOW COVID-19 Ag Card, CareStart COVID-19 Antigen Home Test, BD Veritor System for Rapid Detection of SARS-CoV-2, etc. are the most popular. However, these antigen tests are precise specifically during the first week of illness [10]. The chest XR is paired with the RT-PCR test in clinical practice, providing additional information regarding its severity.On the other hand, a high resolution computed tomography (HRCT) scan is another radiologic option to detect COVID-19, which assists clinicians in identifying the effect of COVID-19 on various organs at different phases of the condition.The management and prognosis of COVID-19 can help with an integrated approach based on first-line CXR and optional use of HRCT [11]. However, there is a scarcity of CT scan facilities and radiologists in remote regions [12,13]. The norm has become for screening methods of COVID-19 and its severity is X-ray imaging together with RT-PCR, because X-ray imaging is rapid, accessible, and less expensive and less radioactively harmful to the human body than Computed Tomography (CT) imaging technology.

If chest X-rays are utilized to test for a diagnosis, expert radiologists must interpret the pictures. However, considering the cryptic nature of visual indicators of COVID-19, lung opacity, and non-COVID viral pneumonia, it can be difficult to diagnose [14].

Moreover, doctors are overwhelmed by the rise of COVID patients, and their workload has grown considerably [15,16]. Considering the surge in COVID-19 cases worldwide and the high workload of doctors and healthcare employees, shortages of radiologists, the development of auto-detection systems based on Artificial Intelligence (AI) to detect COVID is a necessity. A number of methods have been reported for the detection of COVID-19 based on chest-X-rays [17,18] and different deep learning architectures [19], which are approximately 90% or higher in accuracy, but few solutions must be addressed before it is being used in medical environments. The research project in many papers is on the changes and accuracy of network design, but less attention is paid to explaining models, and most of the models are used to enhance the accuracy of models with small data sets. This paper employs three deep learning algorithms for the classificaiton of two classes as well as four classes based on Xception, Resnet50, and VGG19. Through utilizing gradient weighted class activation mapping(GradCam), explainability is achieved, trained and validated on a large dataset considering the back history of publications.

The primary contributions and proposals of this work are, in essence, as follows:

- Three CNN models were developed for COVID-19 mass screening (two classes: COVID positive & COVID negative) from chest X-ray images. Afterward, explainable AI was applied to demystify the black box of models.
- Three additional multi-class models were constructed to diagnose non-COVID, COVID, lung opacity and non-COVID viral pneumonia from chest X-ray radio graphs. Furthermore, explainable AI was used to validate and explain the performance of each generated multi-class model and to demystify the black box of individual CNN layers
- Presented a comprehensive performance study of the proposed binary and multi class systems in terms of the confusion matrix, accuracy, sensitivity, specificity, and F1-score. Additionally, we compared test accuracy for the implemented dual class CNN models for different training input image resolutions and investigated the impact of input image size on the models’ accuracy.

## 2. Background Study

Due to global demand and the advent of Artificial Intelligence (AI), a considerable body of research has evolved to apply Artificial Intelligence (AI) to diagnose different respiratory disorders, particularly those directly related to COVID, utilizing basic XR and CT images. However, the studies in many articles focused on the modifications and accuracy of network design, but less on explaining models, and most models are used to improve the accuracy of models with small data sets. COVID-Net was presented by L. Wang et al., a convolutional neural network [20], in order to detect COVID-19 cases among roughly fourteen thousand chest X-ray images. However, the proposed model managed to gain only 83.5 percent in terms of accuracy. Khan et al. [21] used the transfer learning method on 310 cases of normal pneumonia, 330 cases of bacterial pneumonia, 327 cases of non-COVID viral pneumonia, and 284 COVID-19 pneumonia photos and got 89.5% accuracy. However, as the models employed in this paper used a small number of images, they required detailed analysis. Explainable AI was not implemented to show model efficacy. Y. Oh, S. Park and J. Ye trained the Resnet 18 model and showed explainability [22], but the paper used a low number of images in training and testing and got an accuracy of 89%. Explainability in each layer of the model is desired. T. Ozturk et al. [23] constructed a Darknet framework and trained utilizing only 127 COVID images, and managed to reach 87% accuracy, but model explainability was not explored. A. Altan and S. Karasu [24] trained EfficientNet B and got a promising 99% accuracy, but it was trained with only 219 COVID images, and Explainable AI was not implemented. J. Civit-Masot et al. [25] trained VGG16 with 132 COVID images and got an averageF1 (avgF1) score of 85%, which is not promising in a real clinical scenario due to a low F1 score. Inception v3, InceptionResNetv2, Resnet50 were trained by A. Narin, C. Kaya and Z. Pamuk [26] and got a good accuracy of 98%, which is promising, but those models were trained with 50 normal and 50 COVID images, and the explainability of the model was not depicted. Some methods have focused on creating false data in order to train better models due to the insufficiency of COVID-19 photos. To create fake images, an additional Generative Adversarial Network (GAN) has been used [27]. The results showed that data augmentation boosted accuracy on the VGG16 net from 85% to 95% [28]. J. Arias-Londono, J. Gomez-Garcia, L. Moro-Velazquez and J. Godino-Llorente trained the COVID-net model with large et of images but got 91.53% accuracy [29]. Before a model can be utilized in clinics, it must undergo extensive analysis. Summary results from different letters are given in Table 1.

In summary, numerous recent efforts to transfer learning approaches for detecting COVID-19 from a small dataset have been reported with promising results. Nevertheless, the process required verification on a large dataset. A few models have been developed that have low accuracy and need to be improved. Additionally, the majority of the research does not use explainable AI and does not demonstrate how the model diagnoses COVID-19 from photos. Although a few studies show Explainable AI, they are limited to the final layer of the model only.

In order to address the research’s recurrent issues, we built three transfer learning-based Neural Networks in this study in order to categorize COVID-19 utilizing bigger data sets, exhibiting promising accuracy in an unseen dataset. Likewise, we leveraged Explainable AI to demistify the blackbox of the stated three models and illustrate how the system identifies COVID from X-ray images across all blocks and layers by combining heatmap and original images. Our explainability not only serves as a responsible and transparent audit of our models, but also may assist physicians in improving the screening of COVID-19. Moreover, we trained all three models using images with different input resolutions and found that increasing the image resolution during training improves accuracy in the test condition.Additionally, to address the problems associated with diagnosing patients with non-COVID viral pneumonia and Lung Opacity, we constructed a multiclass model (four classes), validated its accuracy, and explained it using Explainable AI.

## 3. Methodology

In this section, the methodology is depicted in the following order; accumulating data to train the Neural Network, image preprocessing, the experiments and training the Neural Network and evaluation processes.

### 3.1. Dataset

**Figure 1:**
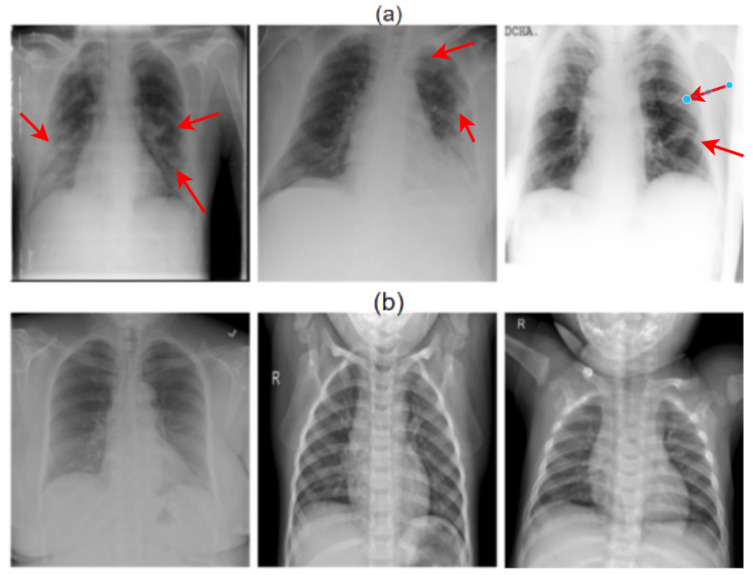
Instances of image data for the two classes of COVID and normal images. Here, (**a**) represents COVID cases and (**b**) depicts normal cases. Here, the red markers indicates the infected laison.

**Figure 2:**
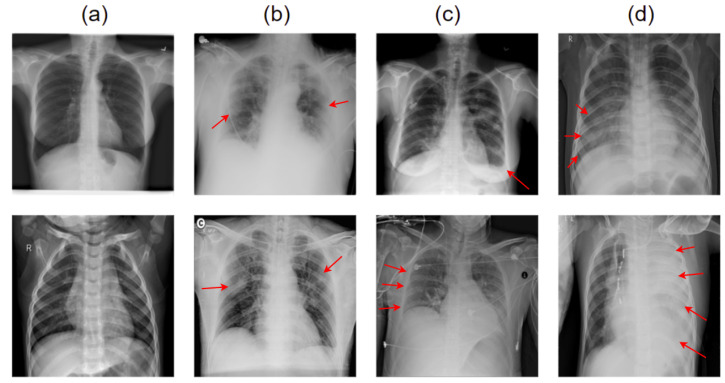
Sample image data for multi class classification to detect (**a**) normal, (**b**) COVID-19, (**c**) lung opacity and (**d**) non-COVID viral pneumonia. Here, the red markers indicates the infected laison.

The study was created utilizing an XR image dataset comprising the PA and AP views (Posterior-Anterior (PA) and Anterior-Posterior (AP) views. This study looked at two different pathological conditions: normal and COVID-19. The majority of the data for this investigation came from Kaggle’s COVID-19-radiography-database [33,34,35,36,37,38,39,40]. The COVID-19-radiography-database was developed by combining different databases [41,42]. For example, images from the COVID-19 DATABASE of the Italian Society of Medical and Interventional Radiology (SIRM) [41], the Novel Corona Virus 2019 Dataset by Joseph Paul Cohen and Paul Morrison, and Lan Dao [42], 43 articles (radiography metadata contains references), Normal and non-COVID viral pneumonia images from the Chest X-ray Images (pneumonia) database. The database includes 3616 COVID-19 positive cases along with 10,192 normal circumstances, 6012 Lung Opacity (Non-COVID lung infection), and 1345 non-COVID viral pneumonia images. Figure 1 depicts two sample instances that contain both COVID and normal classes. Figure 2 shows sample images used for multiclass classification.

As stated before we have considered four types of classes. Among them, COVID-19 is a virus.

#### 3.1.1. COVID-19

COVID-19 is a deadly virus caused by SARS-CoV-2. A minor to acute respiratory illness characterizes the bulk of virus-infected people, who often recover without having to receive special treatment. Nevertheless, few individuals experience severe ailments and require medical attention. Elderly people and people with underlying medical conditions are more prone to have serious medical condition. Anyone who contracts COVID-19 might get critically sick or die at any age.

#### 3.1.2. Non-COVID Viral Pneumonia

Non-COVID viral pneumonia is an infection of the lungs caused by a virus. These viruses usually reside in the upper part of the respiratory system. The issue, though, is when they get into any individual’s lungs. The airways in ones lungs subsequently deteriorate, swell, and flood with fluid. Any element that lowers the effectiveness of ones immune system might make the individual more susceptible to developing pneumonia.

#### 3.1.3. Lung Opacity

Opacity is generally an umbrella term. Any location that selectively attenuates the X-ray beam and looks more opaque than the surrounding area is referred to as having opacity. It’s a general word that doesn’t specify the size or pathologic makeup of the aberration [43]. Any region of the chest radiograph that is whiter than it should be is referred to as such. The lungs generally contain a significant amount of air. When a person gets pneumonia, various substances such as fluids, germs, immune system cells, etc. replace the air in the lungs. Because of this, regions with opacities seem grey when they need to be more black. We realize that the lung tissue in that region is probably unhealthy when we observe them. It includes a variety of other lung infections such as lung cancer, pulmonary edema, pulmonary embolism, etc.

### 3.2. Image Processing

Prior to being fed through neural networks, the X-ray images with the posterior-anterior (PA) and anterior-posterior (AP) views were scaled. We developed six models in total, three of which are utilized to classify normal and COVID pictures. The remaining three models are utilized for multiclass categorization in order to determine whether normal, COVID, lung opacity, and non-COVID viral pneumonia are present in the image.

We carried out two investigations in this paper for binary classification. Data were normalized and scaled to 224 by 224 pixels for the Resnet50 and VGG19 models’ first analysis. According to the standard specification, images for the Xception model are rescaled to 299 by 299 pixels. For our second experiment, we scaled all of the pictures to 512×512 pixels for the three models we developed for two class classifications. The investigation chose 1000 images from positive and 1000 images from non-COVID cases randomly from 3616 COVID-19 positive photographs and 10,192 normal-COVID images from the database. A random 80/20 split was utilized to evaluate the trials’ train and test splits. Moreover, 20% of the train split is utilized as a validation set in order to prevent overfitting. In terms of the second setup models for multiclass, we used 1000 images each of COVID, normal, lung opacity, and non-COVID viral pneumonia instances. The images were resized as per models’ standard requirements, i.e., for Resnet50 and VGG19 input images were resized to 224 by 224 pixels and normalized. For the Xception model, input images were resized to 299 by 299 pixels.

The dataset was also adjusted using Z-normalization. In machine learning methods, standardization helps to stabilize the model while also increasing the training pace. Z normalization is utilized through the following (1).

(1) $$\hat{X}=\frac{X\left[:i\right]-\mu_{i}}{\sigma_{i}}$$

Here, mean value is represented by $\mu_{i}$ and $\sigma_{i}$ denotes standard deviation of the feature.

In this letter, we trained, validated, and tested the collected chest X-ray data in six different Convolution Neural Networks. The Xception, Resnet50, and VGG19 models for both binary class and multiclass were adjusted slightly in the last two layers to achieve regularization.

### 3.3. Neural Network Models

For both the binary class and multiclass set up, three different Convolution Neural Networks were employed to train, validate, and test the accumulated chest X-ray data. The dataset was trained utilizing Xception, VGG19, and Resnet50 models employing Google’s Colab Pro edition hardware consisting of 26.3 GB of system RAM along with 16,160 MB of GPU RAM. Pre-trained weight “image-net” was used to speed up our model’s learning, and the convolutional neural network models were slightly modified along the bottom layers in order to gain regularization. The class imbalance issue in the training data is addressed by applying a weighted categorical loss function. Models are constructed employing Adam optimizer with baseline parameters to ensure computational effectiveness and a quick learning rate. Early stopping has been used with a patience parameter of 15 in order to monitor model’s validation loss and cease training the system once validation loss reaches a stationary state. Our system’s last step was modified utilizing a factor of 0.25 along with patience 15 whenever validation loss hit a plateau. If the model’s performance doesn’t improve following 15 trials, the training is terminated.

**Figure 3:**
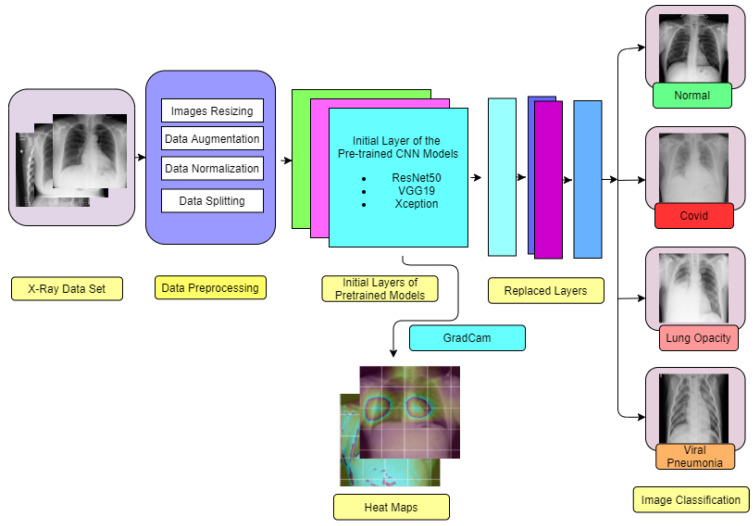
Complete Block Diagram of Experiments. For two class classification only normal and COVID image is detected whereas for multi class problem Normal, COVID, Lung Opacity and non-COVID viral pneumonia is detected.

The models were trained for 200 epochs through a mini-batch size of 16 pictures, and the confusion matrix, resulting ROC curve along with evaluation matrices were created. Furthermore, GradCam is used to build heatmaps in several layers. In order to identify the key areas in the image and predict the pathological state and model interpretability, the input image and heatmap are overlay. Complete system diagram employed in this study is depicted in Figure 3.

#### 3.3.1. VGG19

**Figure 4:**
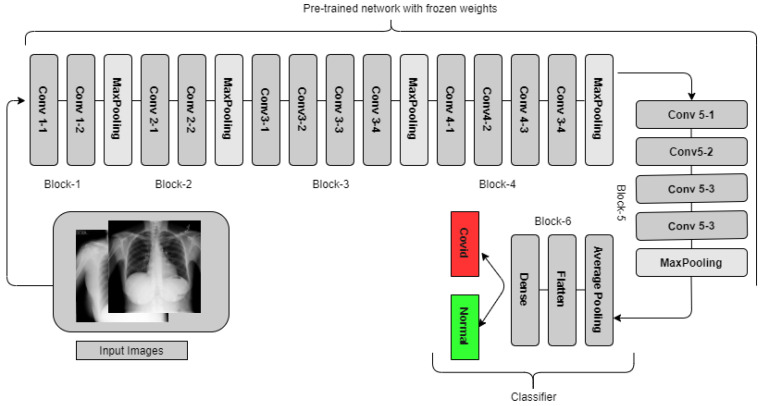
VGG19 in our classification experiments. For the four class problems, Normal, COVID, Lung Opacity, and non-COVID viral pneumonia were detected, whereas for two classes, only COVID and normal images were detected.

In the Image Net Large Scale Visual Recognition Challenge (ILSVRC) in 2014, VGGNet [44] was a neural network that did exceptionally well. It came in first place for image localization and second place for image classification. Figure 4 shows VGG19 in our classification experiment, which consists of 16 convolution layers, 5 Max-pooling layers, one average pooling, flattening, and a dense layer. VGG CNN has six major blocks which make the network deep, utilizing small 3 × 3 filters, with a stride of 1, and the same padding for the Conv Layers and 2 × 2 filters with a stride of 2 for the Maxpooling/Downsapling layers. In our tailored VGG19 network, we froze the weight up to the max-pooling layer of the conv5 block, and then we added one average pooling, flattening, and dense layer to finetune the model.

#### 3.3.2. Resnet50

ResNet [45] has emerged as a ground-breaking deep neural network (DNN) model for computer vision problems. It made its debut in 2015 when it won the ImageNet [46] competition.

**Figure 5:**
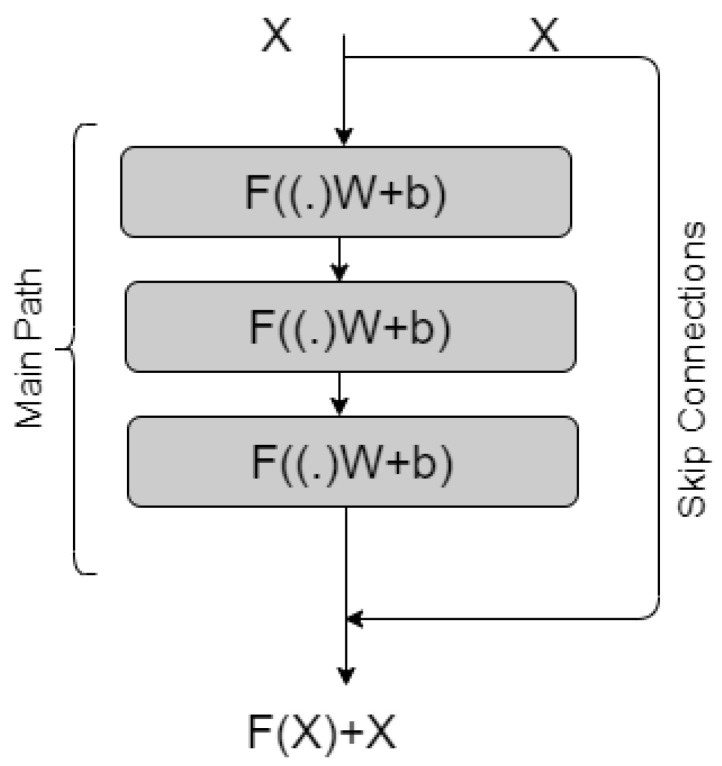
One Resblock in resnet50 with main and skip connection.

In most situations, DNNs outperform neural networks (NN) with fewer layers. However, training a massively stacked NN is notorious for its vanishing gradient issue, which causes model performance to deteriorate. Identity shortcut connections or skip connections that bypass one or more layers—have been used to overcome this issue in ResNet. The use of residual blocks, which consist of the main path and identify shortcut links, helps to solve the vanishing gradient problem, which is shown in Figure 5.

The primary path consists of a sequence of Neural Networks, whereas the second path, known as skip connections, is a straight path from input to output. Skip links connect to the output of the network directly and the network provides output as F(X)+X. The Skip connection addresses the gradient vanishing problem associated with conventional neural networks.

**Figure 6:**
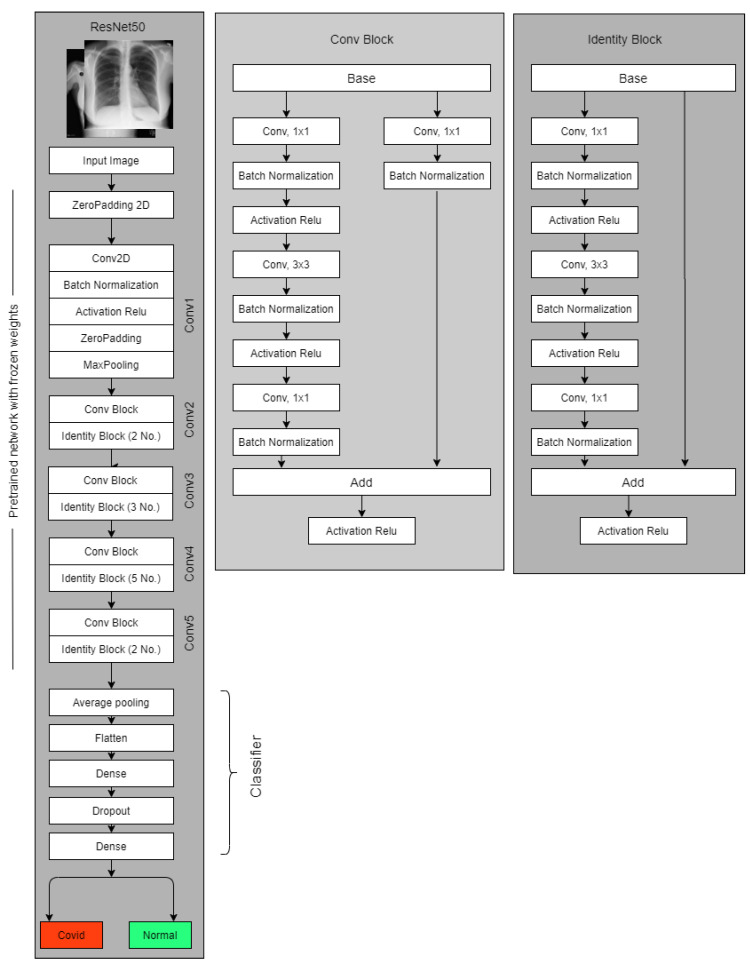
Modified Resnet50 to Classify COVID and Normal images. For the four classes, similar architecture is applied and the final dense layer has an output size of four to detect normal, COVID, Lung Opacity and non-COVID viral pneumonia images.

We used the rasenet50 version of Resnet and tweaked it somewhat in the final stages. We utilized the weight of the image-net to create a classification detector for COVID and normal instances by freezing the weight of the top layers.Our proposed structure for the ResNet-50, which is used to categorize chest X-rays, is shown in Figure 6.

On top of the pre-trained model, more layers are added. To classify images, an average pooling layer with a pool size of (4, 4), a flattening layer, a dense layer with Relu activation, a dropout layer with a dropout probability of 50% drops 50% of the parameters randomly and reduces overfitting, and finally, a dense layer is used.

#### 3.3.3. Xception

Xception [47], a modified version of Inception-v3, is a Depthwise Separable Convolutions-based deep neural network architecture which Google researchers developed. Each input stream is processed through a single convolutional filter during a convolution process known as depthwise convolution. A type of convolution known as pointwise convolution uses a 1 × 1 kernel that iterates over each point. The depth of this kernel is equivalent to the quantity of channels in the source image. To generate depthwise-separable convolutions, a pointwise convolution is combined with a depthwise convolution.

**Figure 7:**
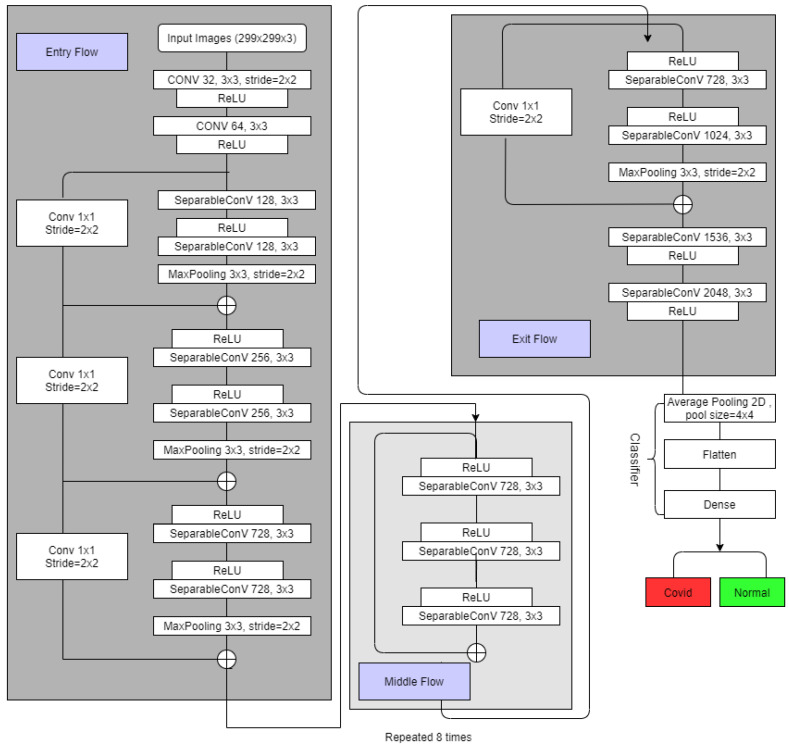
Modified Xception Network to Classify COVID and Normal images. For the four classes, similar architecture is applied and the final dense layer has an output size of four to detect normal, COVID, Lung Opacity and non-COVID viral pneumonia images.

In the original depthwise separable convolution, a pointwise convolution comes after the depthwise convolution, while in the modified depthwise separable convolution, a depthwise convolution comes after the pointwise convolution. The Xception model used modified separable convolutions. Our tweaked Xception model is depicted in Figure 7.

As in the figure, Separable Convolutions are the modified depthwise separable convolutions, and there are residual connections in the middle flow. In the Xception model, the data initially passes via the input flow, then eight times through the middle flow, and lastly through the exit flow. For the system, we employed pre-trained “image-net” weight, and we tweaked a couple of the model’s final layers to gain regularization. Average Pooling layer with a pool size of 4 by 4, a flattening layer, and a dense layer is added to classify images.

### 3.4. GradCam

Grad-CAM, also known as gradient-weighted class activation mapping, is a method for identifying the areas of an image that are crucial for a certain classification decision. Convolutional neural networks (CNNs) are frequently employed for tasks in computer vision including, where it is utilized to interpret predictions. The process entails computing the gradients of the last convolutional layer’s output with respect to the input picture and then adjusting the gradients to account for the relative weights of each feature map in the final judgment. To highlight the areas that are crucial for the projected class, the generated heatmap is superimposed over the original image. In order to comprehend the internal representations acquired by CNNs and also to enhance the interpretability of these models, Grad-CAM has been widely employed in the area of computer vision. It employs gradients from any target idea and feeds them into the final convolutional layer to create a coarse localization map that highlights key areas in the picture that are crucial for concept prediction.

### 3.5. The Experiments

Our tweaked and proposed three models were utilized in this investigation for two separate experiment setups.

#### 3.5.1. Two Class Setup

In two class experiment setup we utilized all the three proposed model utilizing last dense layer with an output size of 2. we did two investigation with this experiment setup.

In the first investigation, models were trained with standard image requirements as per Xception, VGG19 and Resnet models. The standard inut size requirements for Xception, VGG19 and Resnet are 299×299, 224×224 and 224×224 pixels respectively, for each of the three algorithms. In the second investigation, we first resized the input photos to 512×512 pixels, and then we trained all of the proposed models using the resized input images.

For both the investigation, model performances are evaluated using unseen data. The performance of both experimental setup with three proposed model is evaluated using data that has not been observed by the model while training.

#### 3.5.2. Multi/Four class Setup

In multi class experiment setup we used all the three proposed model utilizing last dense layer with an output size of 4. The Xception, VGG19, and Resnet models were trained in the field of standard picture needs. The typical criteria for inut size are 299×299 for Xception, 224×224 for VGG19 and Resnet accordingly.

### 3.6. Evaluating Model Performances and Deep Layer Feature Investigation

We evaluated accuracy, recall or sensitivity, precision or also known as Positive Predictive Value (PPV), F1 score, along with specificity in order to asses the performance of our employed algorithms for classifying X-ray pictures as per (2), (3), (4), (5), and (6). Precision [48] is defined as the ratio of correct positive identifications relative to all positive identifications. A low precision will result in a significant number of false positives, meaning patients will be mistakenly categorized as having a certain condition. The recall [48] is the number of true positives over the number of true positives plus the number of false negatives. Recall takes the false negative rate into consideration. Incorrect diagnosis may occur due to low recall rate. The F1-score (6) is the harmonic mean of precision and recall. A high F1 score is desired in the classification task.

(2) $${\mathrm{Accuracy}}_{i}=\frac{{\mathrm{TP}}_{i}+{\mathrm{TN}}_{i}}{{\mathrm{TP}}_{i}+{\mathrm{TN}}_{i}+{\mathrm{FP}}_{i}+{\mathrm{FN}}_{i}}$$

(3) $${\mathrm{Precision}}_{i}=\frac{{\mathrm{TP}}_{i}}{{\mathrm{TP}}_{i}+{\mathrm{FP}}_{i}}$$

(4) $${\mathrm{Sensitivity}}_{i}=\frac{{\mathrm{TP}}_{i}}{{\mathrm{TP}}_{i}+{\mathrm{FN}}_{i}}$$

(5) $${\mathrm{Specificity}}_{i}=\frac{{\mathrm{TN}}_{i}}{{\mathrm{TN}}_{i}+{\mathrm{FP}}_{i}}$$

(6) $$F1\_{\mathrm{score}}_{i}=2\times \frac{{\mathrm{Precision}}_{i}\times {\mathrm{Sensitivity}}_{i}}{{\mathrm{Precision}}_{i}+{\mathrm{Sensitivity}}_{i}}$$

where,

- i = COVID and Normal for classification problem.
- TP = True Positive
- FN = False Negative.
- TN =True Negative

**Figure 8:**
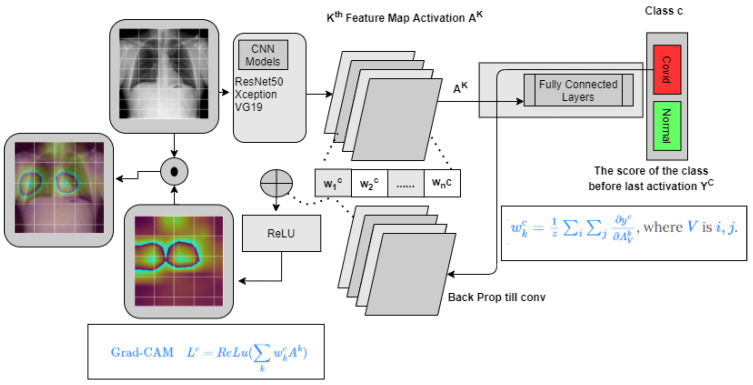
The complete process for Gradcam analysis for COVID and non-COVID classes. For the four classes, the same architecture is applied except the output class is COVID, Normal, Lung Opacity, and non-COVID viral pneumonia.

This study employed the gradient weighted Class Activation Mapping (GradCAM) [49] technique to visualize the input regions significant for model predictions and to visualize heatmaps in three distinct CNN network layers to simplify the model performances. The comprehensive Gradcam analysis method is shown in Figure 8. By visualizing heatmap, it is often possible to depict why the model concluded a particular class. Prior to developing the class prediction of the image, we first sent an image through the model to produce a prediction. After that, we computed the gradient of the class with respect to Feature Map activation *A*${}^{k}$

(7) $$A^{k}=\frac{\partial y^{c}}{\partial A_{ij}^{k}}$$

To derive the neuron significance weights, these gradients flowing back are global-average-pooled across the width and height dimensions (indexed by *i* and *j*, respectively).

(8) $$w_{k}^{c}=\frac{1}{Z}\sum_{j}\sum_{i}\frac{\partial y^{c}}{\partial A_{ij}^{k}}$$

We measure Grad Cam utilizing equation x.

(9) $$L_{\mathrm{GradCAM}}^{c}=ReLU\left(\sum_{k}w_{k}^{c}A^{k}\right)$$

By overlaying the input image with heatmap, we developed visualization. For the binary class, this letter examined the GradCam visualization for all the Xception model layers and compared heatmap across all the models to find better efficacy and explain which part of the features affecting model to decide a particular class. First, we analyzed different layers and different blocks of a model to find which regions affect categorisation using GradCam. Second, we compared the final convolution layer of activation maps of employed CNN models to demystify ita performances.For the multiclass classification we analyzed last CNN layers heatmap for the three models and concluded with comparing the same.

## 4. Result Analysis

The results of the experiments are broken down into two sections. In the first phase, results were analyzed statistically, and in the second phase, feature extraction in different layers of different models was visualized using GradCam and alalyzed why our model came to the conclusion of detecting COVID and non-COVID images.

### 4.1. Statistical Analysis

We assessed the model’s performance using a separate test set that models were not exposed to during training. The models were evaluated statistically by computing the test precision, recall, F1-score (F1), accuracy (Acc), Positive Predictive Value (PPV), and the Area Under the ROC Curve (AUC).

**Figure 9:**
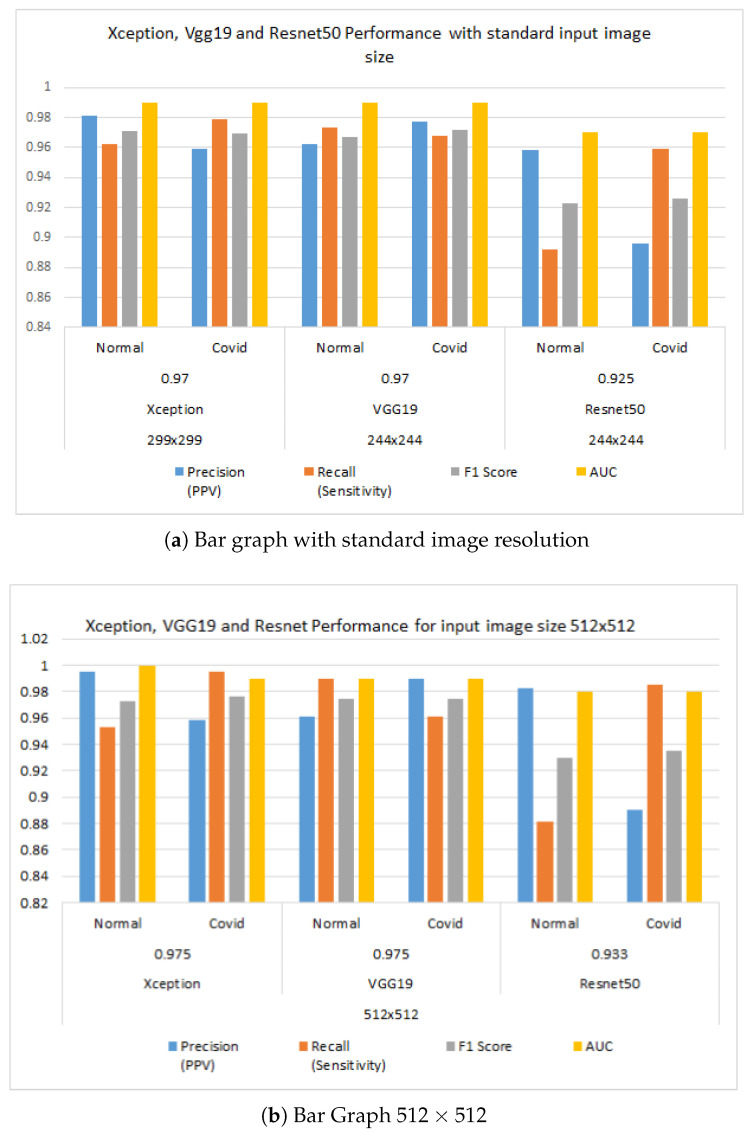
Precision, recall, F1 score and AUC bar bar chart comparison for three models: Xception, VGG19 and Resnet50 while training the model with standard input image size and 512×512 input image size. Precision, recall, F1 score and AUC bar bar chart for three models when model is traned with standard image size (**a**). Precision, recall, F1 score and AUC bar bar chart for three models when models are trained with the images size of 512×512 (**b**). Resnet performs poorly, and with the increase in image size, accuracy increases.

The effectiveness of the three employed CNN network is examined for a two-class setting is summarized in Table 2. Performance is also shown for the implemented three networks for two different input image sizes. Similarly, Figure 9 shows the performance comparison of all three models at normal picture size as per model’s requirement and 512×512 input images.

From Table 2, we can examine the accuracy of the Xception and VGG19 is 97% while training the model with standard image size requirements as per model, while the Resnet50 provides 92.5% accuracy. The F1 score for detecting normal and COVID images for Xception and VGG19 is 0.971, 0.969, 0.967, and 0.972 respectively, where as for the resnet model, it is 0.923 and 0.972. Clearly, Xception and VGG are outperforming the Resnet 50 considering accuracy and F1 score. The sensitivity to detecting COVID is higher than the other two models and it is 97.9%, where high precision of 97.7% is observed in VGG19 for the COVID class. For the normal class, recall is highest in VGG 19 and precision is highest in the Xception model.For the 512×512 image size, again, accuracy is higher in Xception and VGG19 and both provide an accuracy of 97.5%. The F1 score for COVID and the normal class for the Xception model is 0.976 and 0.973, where as in the VGG19 model, it is 0.975 and 0.975. So, it can be concluded that training with increased image size improves the accuracy and performance of the model. This may be because, while resizing to a lower resolution, we may lose some vital information from the image which may take part in decision making processes. Figure 9 shows two bar graphs to compare model to model performance at different image input resolutions while training. It is evident that Resnet is performing poorly compared to VGG19 and Xception. It is also evident that training with increased image resolution helps to achieve better performance of the models.

**Figure 10:**
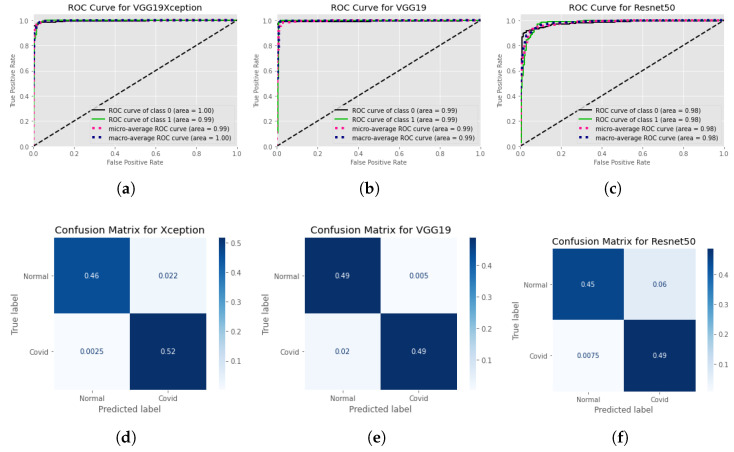
ROC curves and confusion matrices for each of the models, considering each of the classes individually while training the model with 512 × 512 input image resolution. ROC curves are at the top Normalized confusion matrices are at the bottom. Left: Xception model’s ROC curve and confusion matrices. Center: Confusion matrices and the ROC curve of the VGG19 model. And finally at right: Resnet model’s ROC curve and confusion matrices. (**a**) ROC curve for Xception model at input image resolution 512×512, (**b**) ROC curve for VGG19 at input image resolution 512×512, (**c**) ROC curve for Resnet50 at input image resolution 512×512, (**d**) Confusion Matrix for Xception model at input image resolution 512×512, (**e**) Confusion Matrix for VGG19 at input image resolution 512×512, (**f**) Confusion Matrix for Resnet50 at input image resolution 512×512.

Figure 10 shows the ROC curves per class for each model and the accompanying confusion matrices at standard image size as input during the training of models, and Figure 10 shows the same for the models when trained at 512×512 input images. In addition to the previous finding, it is undeniable that training models with higher picture quality results in enhanced performance of the models after they have been trained.

**Figure 11:**
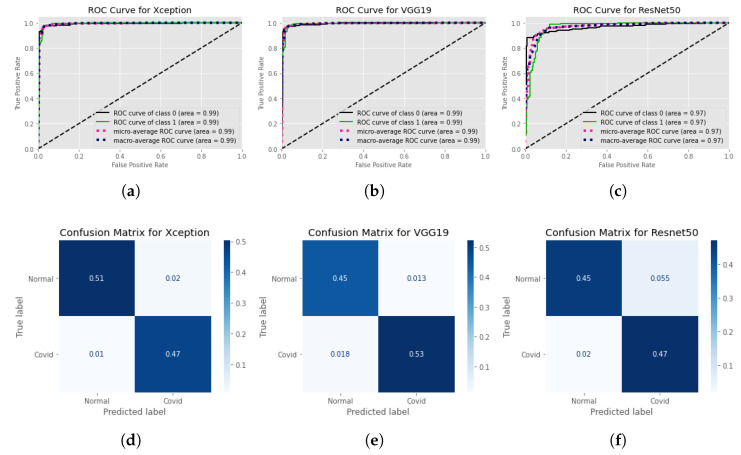
ROC curves and confusion matrices for each of the models, considering each of the classes individually while training the model with standard image size. ROC Curves are presented at Top. Normalized confusion matrices are at Bottom. Left: Xception model’s ROC curve and confusion matrices. Center: Confusion matrices and the ROC curve of the VGG19 model. And finally at right: Resnet model’s ROC curve and confusion matrices. (**a**) ROC curve for Xception model at input image resolution 299×299, (**b**) ROC curve for VGG19 at input image resolution 224×224, (**c**) ROC curve for Resnet50 at input image resolution 224×224, (**d**) Confusion Matrix for Xception model at input image resolution 299×299, (**e**) Confusion Matrix for VGG19 at input image resolution 224×224, (**f**) Confusion Matrix for Resnet50 at input image resolution 224×224.

**Figure 12:**
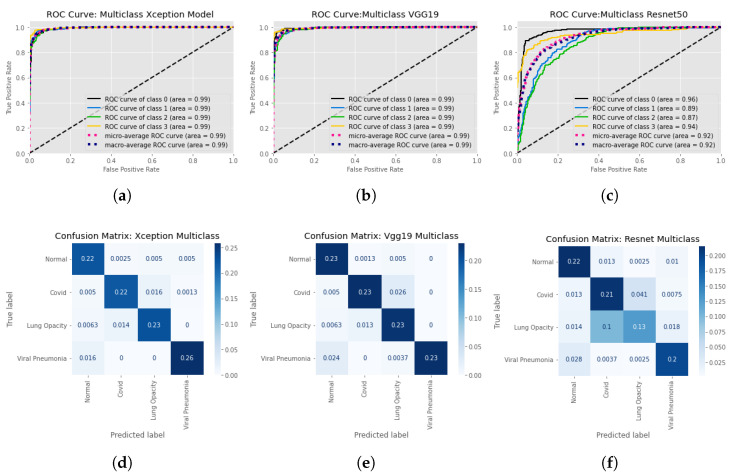
ROC curves and confusion matrices for Multi Class models, considering each of the classes individually while training the model with standard image size. ROC curves are at the top Normalized confusion matrices are at the bottom. Left: Xception model’s ROC curve and confusion matrices. Center: Confusion matrices and the ROC curve of the VGG19 model. And finally at right: Resnet model’s ROC curve and confusion matrices. (**a**) ROC curve for Multi Class Xception model at input image resolution 299×299, (**b**) ROC curve for Multi Class VGG19 at input image resolution 224×224, (**c**) ROC curve for Multi Class Resnet50 at input image resolution 224×224, (**d**) Confusion Matrix for Multi Class Xception model at input image resolution 299×299, (**e**) Confusion Matrix for Multi Class VGG19 at input image resolution 224×224, (**f**) Confusion Matrix for Multi Class Resnet50 at input image resolution 224×224.

The effectiveness of the three employed CNN network is examined for both binary and multi class classification. Figure 11 denotes the findings of our training for binary classification and multi-class arrangement is summarized in Table 3 and Figure 12. We can see the Xception model is outperforming VGG19 and Resnet50 and giving accuracy 93%. F1 score for Normal, COVID, Lung Opacity, and non-COVID viral pneumonia is 0.92, 0.92, 0.96 respectively. VGG19 is quite close performance wise and gives 92% accuracy.

### 4.2. Model’S Explainability and Interpretability

It is commonly feasible to show why the model determined a specific class using a heatmap. We qualitatively examined network-identified regions of interest using Grad-CAM activation maps and by displaying heatmaps at different layers of three proposed networks. To anticipate the pathological condition and model interpretability, and to further analyze, the input data along with its heatmap are superimposed to locate the critical spots in the image. Although the last CNN layer is generally used in GradCAM literature, we visualized all the network layers to study the learning process of the models, since the last layer contains high-level information. In this letter, we utilized the GradCAM approach to (1) visualize and compare the different layers of a model to identify the model’s decision processes and (2) identify the network sections that have the most significant impact on categorization. (3) to compare activation maps of three CNN models’ last convolution layer to demystify all the model performances. For the Xception model, we used GradCAM to display the activation maps for all the CNN blocks for COVID and non-COVID class images. We visulaized the activation map for all the layers in the same block for the Xception model. Furthermore, we implemented expainable AI to the Resnet50 and VGG19 models and compared the activation maps to see which model should perform better in clinic conditions. We approached GradCam analysis for four class classification to show the last conv layer output of the Xception models since the accuracy of Xception model is higher than the VGG19 and Resnet50.

**Figure 13:**
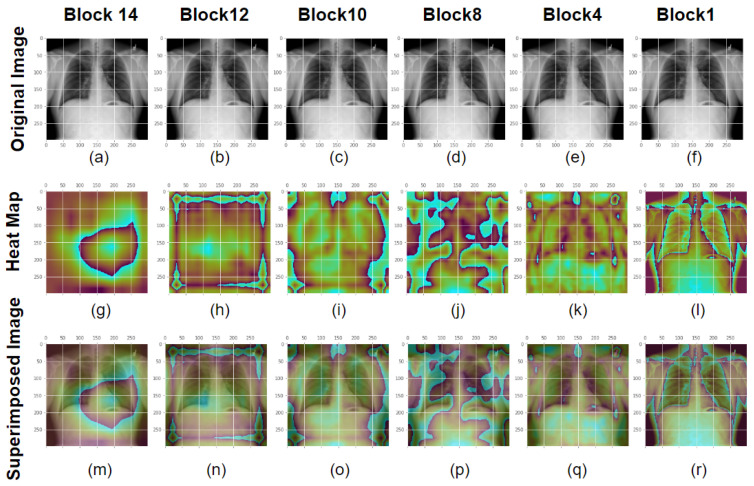
GradCam Analysis for the Xception model for normal class images at different layers. Chest X-ray image from the first row or in images (**a**–**f**) is in the normal class. The second row or images (**g**–**l**) depicts the X-ray images after GradCam activation mapping. Activation map and an X-ray picture are overlaid in the third row or images (**m**–**r**). The block14 separation convolution 2 from the Xception model is displayed in the first column along with the input image, heatmap, and overlay image. The second, third, fourth, fifth, and sixth columns are for Block 12 sepconv2, Block 10 sepconv2, Block8 sepconv2, Block4 sepconv2, and Block1 conv1 respectively. Block 14 detects high-level features and impacts decision making, where as block 1 detects lower-level features such as colors and edges.

**Figure 14:**
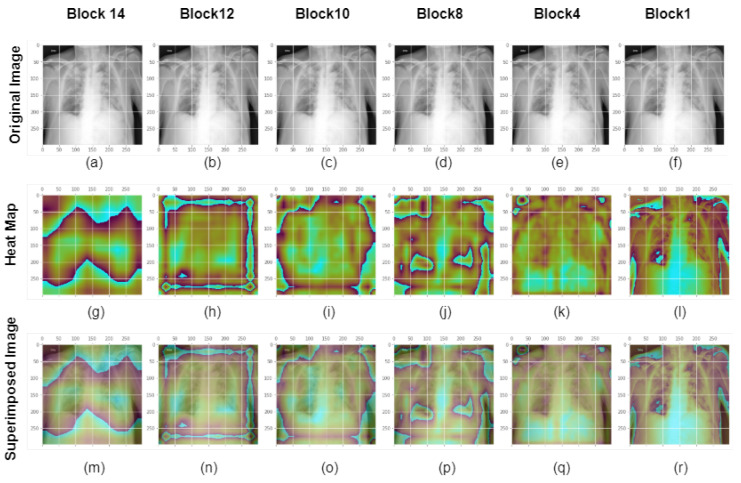
GradCam assessment of Xception-modeled class images at different levels. A chest X-ray picture from the COVID class is displayed in the first row or in images (**a**–**f**). The second row or images (**g**–**l**) displays the GradCam activation mapping for the X-ray picture. Images (**m**–**r**) or the third row: An activation map with an X-ray image overlaid to display the superimposed image. The block14 separation convolution 2 of the Xception model’s original picture, heatmap, and overlay image are shown in the first column. The second, third, fourth, fifth, and sixth columns correspond to Block 12 sepconv2, Block 10 sepconv2, Block 8 sepconv2, Block 4 sepconv2, and Block 1 conv1. Block 14 detects high-level features and influences decision-making, whereas block 1 detects lower-level features like colors and edges.

Figure 13 depicts the visualization results for the Separation Convolution layers in Block 14, 12, 10, 8, 4 and 1 for the Xception model for a ground truth normal image. From the original X-ray images, heatmap, and superimposed images, we can visualize that Separation Convolution block 14 has the most significant impact on image classification as it can see the high-level complicated features. Block 14 builds up features by combining all the features that were detected in the early layers. On the other hand, block 1 just impacts the model performance by detecting low-level features such as edges and colours. The Deep layers feature mainly contributed to better offering an explanation of the failure or success of a deep learning network in a particular decision. In this case, the Xception model detected the image truly and detected it as a class of image which is normal. Similarly, Figure 14 shows that X-ray images with ground truth COVID diagnosis has a comparable impact, indicating that Block 14 strongly influences finding classification areas. It can be seen that in the last block convolution layer, the model is looking into the chest region, which is the region of interest for detecting COVID, and the model almost sees all the regions of the COVID affected area.The Xception model was flawless in this case, predicting it. In summary, the last block contributes more to the classification than the whole block.

**Figure 15:**
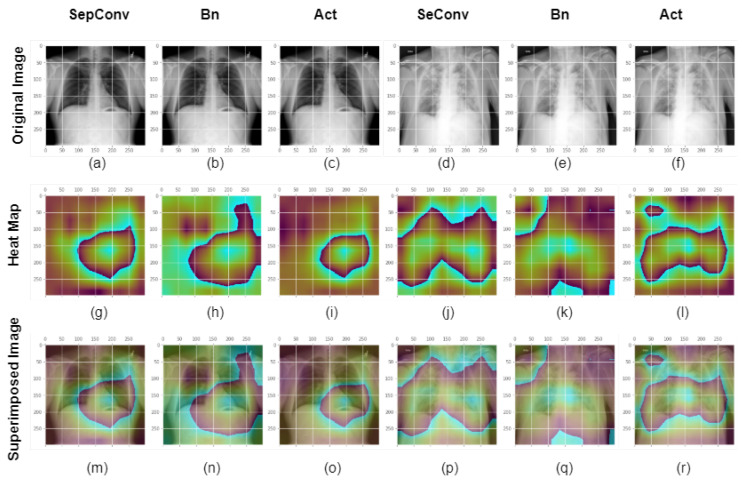
GradCam analysis utilizing the Xception model of COVID and normal class images at several levels in a single block. Chest X-ray image from the COVID and normal classes are presented in the first row or in images (**a**–**f**). Images (**g**–**l**) or the second row depicts the xray image’s GradCam activation mapping. The X-ray image in the third row or images (**m**–**r**) is positioned on an activation map to display the superimposed image. The input image, heatmap, and overlay image of the block14 separation convolution 2, batchnormalization, and ReLu activation layers of the Xception model for a normal class image are shown in the first three columns. The original picture, activation mapping, and overlaid images of block14 separation convolution 2, batchnormalization, and ReLu activation layer for a COVID class image for the Xception model are shown in the latter third of the column. The Activation layer contributes more to the decision making process.

In the search for determining which layer in a convolutoin block provides more decisiveness, for COVID and normal classes, we checked the sep convolution layer, batchnormalization (bn) layer and activation (act) layer heatmap along with the original and superimposed image, shown in Figure 15. The first row displays COVID and normal chest xray images. In the second line, the xray image’s GradCam activation mapping is displayed. The third row displays superimposed images. The input image, heatmap, and overlay image of the block14 separation convolution 2, batchnormalization, and ReLu activation layers of the Xception model for a normal class image are shown in the first three columns. The original image, activation mapping, and overlaid images of block14 separation convolution 2, batchnormalization, and ReLu activation layer for a COVID class image for the Xception model are depicted in the bottom third of the column. The activation layer model there observes a more narrow region in search for COVID and normal classes. Although the convolution layer watches the spread region, the activation contributes more to the decision making process. Since the activation layer is closest to the output, the activation layer must detect relevant features and in our Xception model it does the same.

**Figure 16:**
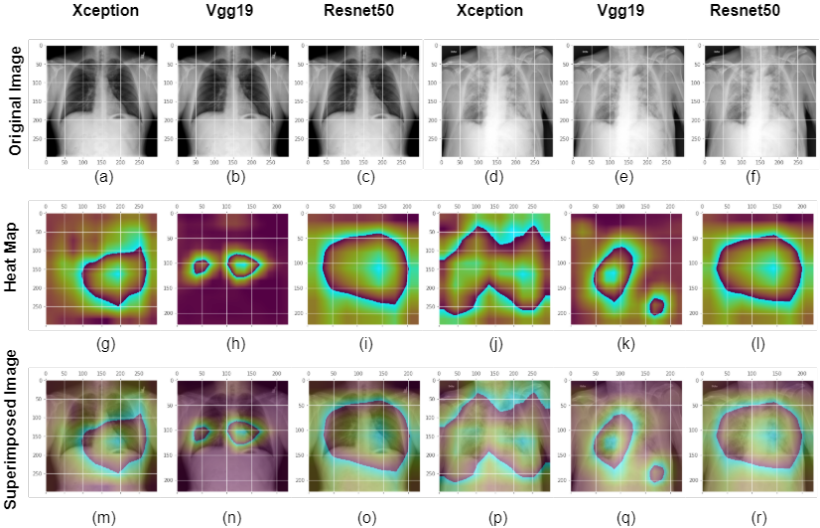
GradCam assessment of COVID and normal class images at the final convolution layer in the Xception, VGG19, and Resnet models. An X-ray image of the chest from the COVID class is depicted in the first row or in images (**a**–**f**). Images (**g**–**l**) or the second row depicts the GradCam activation mapping for the X-ray picture. Third row or images (**m**–**r**): An activation map with an X-ray image overlaid to display the superimposed image. The original picture, heatmap, and overlay image from the final convolution layer of the Xception, VGG19, and Resnet50 models for a normal class image are shown in the first three columns. For a COVID class image, the final convolution layer of Xception, VGG19, and Resnet50 is shown in the last third column along with the original picture, activation mapping, and overlay image. The final convolution layers for the Xception, VGG19 and Resnet models are block14 sep conv 2, block5 conv 4 and conv5 block 3_3 conv respectively.

In the final stage of our model visulization for two class classification, deep layer feature investigation, and model explainability, We examined the heatmap of the most recent convolution layer for each model we had used. Figure 16 displays the GradCam analysis of COVID and standard class pictures at the final convolution layer in the Xception, VGG19, and Resnet models. A chest X-ray picture from the COVID and normal classes is displayed in the first row. The second row displays the GradCam activation mapping for the X-ray picture. The usperimposed image in the third row is displayed using an X-ray image put on an activation map. The original picture, heatmap, and overlay image from the Xception, VGG19, and Resnet50 models for a normal class image are shown in the first three columns. The final convolution layer of Xception, VGG19, and Resnet50 for a COVID class image appears in the bottom third of the column along with the original image, activation mapping, and overlay image. The final convolution layers for the Xception, VGG19 and Resnet models are block14 sep conv 2, block5 conv 4 and conv5 block 3_3 conv respectively. From the image shown, the VGG19 and Xception models are more looking into the high lever features in search of desired regions. For normal class, all three models look into the desired region and all three models predict it perfectly. However, in COVID class VGG19 misclassified the image and from the heatmap we can see that although it is watching in the chest region, it came into prediction by watching in the other portion rather than the chest.

**Figure 17:**
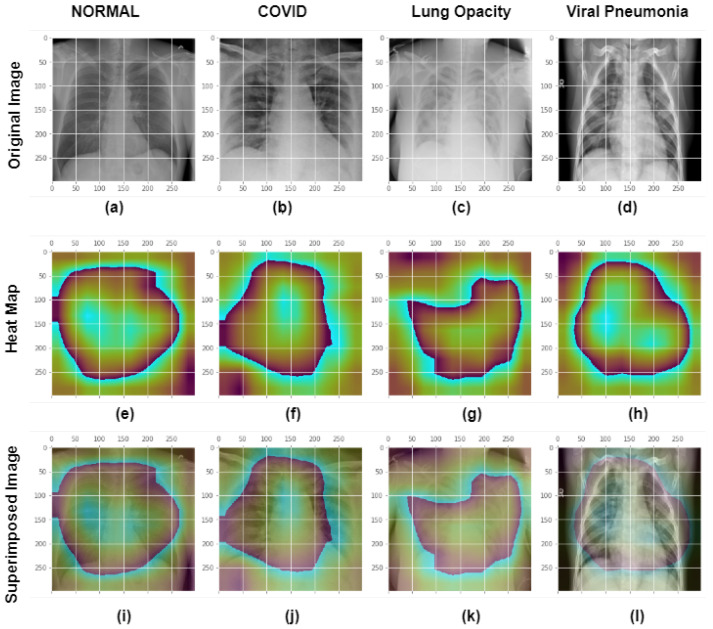
GradCam analysis of four class photos at the final convolution layer in the Xception model. First row or images (**a**–**d**): shows chest xray images of different classes. Images (**e**–**h**) or the second row depicts the GradCam activation mapping for the X-ray picture. Images (**i**–**l**) or Third row: An activation map with an X-ray image overlaid to represent the superimposed image. The Normal class’s original picture, heatmap, and overlay image are shown in the first column of the Xception model’s final convolution layer. The original picture, activation mapping, and overlay image for the COVID, Lung Opacity, and non-COVID viral pneumonia classes are shown in the second, third, and fourth columns, respectively. All the images were correctly classified in this case.

**Figure 18:**
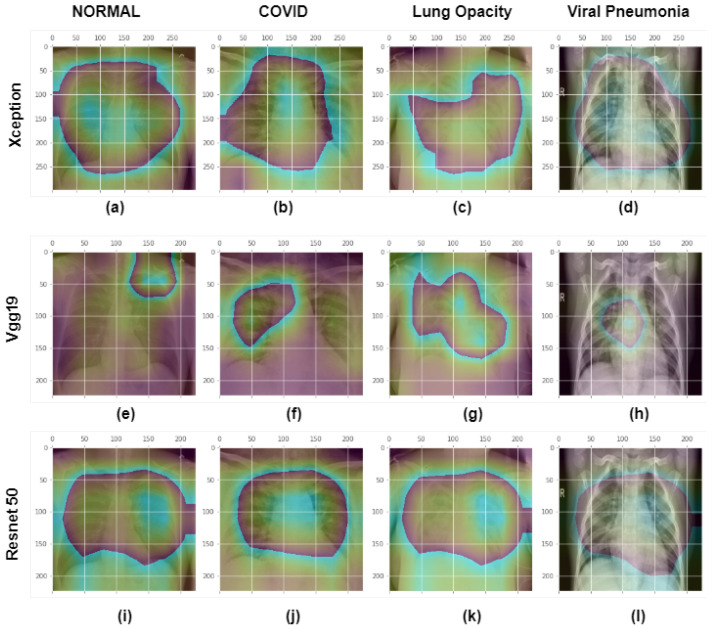
GradCam analysis of four class photos at the final convolution layer in the Xception, VGG19, and Resnet50 models. First row or images (**a**–**d**): shows superimposed images of different classes in the last layer of the Xception model. Images (**e**–**h**) or Second row: shows superimposed images of different classes in the last layer of the VGG19 model. Images (**i**–**l**) or Third row: shows superimposed images of different classes in the last layer of the Resnet model. The first, second, third, and fourth columns display superimposed images of normal. COVID, Lung opacity and non-COVID viral pneumonia classes. Xception and Resnet models classified all the images correctly, but VGG19 misclassified Normal and non-COVID viral pneumonia classes.

GradCam analysis of four class photos is shown in Figure 17 and Figure 18. Explainable AI at the final convolution layer in the Xception model is visualized in Figure 17. First row: shows chest xray images of different classes. The second row displays the GradCam activation mapping for the X-ray image. Third row: An activation map with an X-ray image overlaid to display the superimposed image. The Normal class input picture, heatmap, and overlay image are shown in the first column of the Xception model’s final convolution layer. The second, third, and fourth columns display the original image, activation mapping, and superimposed image for the COVID, Lung Opacity, and non-COVID viral pneumonia classes respectively. All the images were correctly classified in this case and we can see the model is watching the region of interest to differentiate Normal, COVID, Lung Opacity and non-COVID viral pneumonia classes. From Figure 18 it is possible to visualize that VGG19 misclassified COVID and non-COVID viral pneumonia classes as the last layer is watching some other points rather than the desired region. However, the Xception and Resnet models detected image classes perfectly in this case.

### 4.3. Discussion

We have utilized three different pre-trained models for our training that are Xception, VGG19 and ResNet50. For binary classification we considered two type of image sizes that are 299 × 299 and 512 × 512. With 512 × 512 image sizes, we have managed to reach 97.5%, 97.5%, and 93.3% for Xception, VGG19 and ResNet50 respectively. Figure 10 represents the ROC and the confusion matrix for each of the trained model for binary classification that are trained with 512 × 512 image sizes. Based on the performance metrics, the Xception model is shows more superiority because of its precise feature extraction mechanism in entry, middle and exit flow. From the confusion matrix we can observe that it can almost flawlessly classify COVID-19 images. Only 0.0025% of COVID-19 images are misclassified as normal X-ray images. On the other hand, 0.022% normal images are misclassified as COVID-19 images. With 299 × 299 image size input our Xception, VGG19 and ResNet50 model gained 97%, 97%, and 92.5%. Figure 11 depicts the ROC curve and the confusion matrix for the trained models with 299 × 299 image size. Here Once again, Xception Model turned out to be the most efficient model. It can classify COVID-19 images more correctly than any other models. However, VGG19 excelled in classifying normal images. It has the lowest false positive value in terms of classifying normal X-ray images.

After finalizing our binary classification model, we moved on to multi-class classification with VGG19, Xception, and ResNet50. Among the utilized models ResNet50 scored only 75% while VGG19 scored 92% and Xception Score 93%. Figure 12 denotes the generated ROC curve and confusion matrix from our trained multi-class models. Figure 12d states that our trained Xception model is superior in classifying non-COVID viral pneumonia images. However, the chances of COVID-19 images being misclassified with lung opacity are higher. In terms of VGG19 there are fewer false positive values. Although, it can classify non-COVID viral pneumonia and normal images more accurately it tends to misclassify COVID-19 images as lung opacity class. Although, both the model reached almost similar accuracy, it’s fair to state that Xception is a more efficient model.

## 5. Conclusions

This article proposes a comprehensive CNN-based transfer learning approach for classifying between non-COVID viral pneumonia, lung opacity, COVID-19 and normal X-ray images. Throughout the manuscript, we have presented six framework whereas the first three models automatically recognize COVID-19 and the other three models classify between normal, COVID-19, lung opacity and non-COVID viral pneumonia images. Our experiment was split into two segments. In the initial segment, we only consider the binary classification of COVID-19 and in final segment we conduct multi-class classification. We have considered a varaity of image size while conducting our experiment and it is concluded that image input of 512×512 pixels delivers greater accuracy, which might be because image resizing loses some essential information from photos, lowering accuracy. For binary classification between COVID-19 and normal X-ray images, with standard data input the accuracy of Xception, VGG19, and Resnet is 97%, 97%, and 92.5%, respectively. With 512 by 512 pixels, the accuracy improved to 97.5%, 97.5%, and 93.3%. Each of the model managed to gain satisfactory auc, precision, recall and f-1 score which indicates none of the trained model is biased towards any particular class. Additionally, for multi-class classification, Resnet50 reached an accuracy of only 75% whereas VGG19 reached 92% and Xception gained 93% with satisfying precision, recall, auc and f-1 score. Furthermore, we have utilized Explainable AI that introduces interpretability to our models. This section revealed that, although almost both Xception and VGG-19 model managed to acquire moderate score, Xception was the most superior in terms of identifying the precise features responsible for a model’s prediction.

Even though we were able to achieve an accuracy of 93%, it may still be unstable in everyday use as the patients are directly impacted by the predictions made by the model, and even a single misclassification might have serious consequences. Thus, in the future, we intend to further improve the models accuracy and want to utilize various transformer-based frameworks that can reach higher accuracy. Moreover, for the multi-class classification we have only considered COVID-19, non-COVID viral pneumonia, normal and lung opacity X-ray images. However, lung opacity includes a variety of other lung infections such as lung cancer, pulmonary edema, pulmonary embolism, etc. We aim to add additional classes in our future model that can classify X-ray images beyond just lung opacity. Furthermore, since medical data requires an extra layer of privacy we plan on developing a federated learning based model.

## Figures and Tables

**Table 1:** Overview of the existing related studies (Here, Acc = Accuracy, avF1 = Average F1-score, Se = Sensitivity, SP = Specificity).

Reference Number	Number of Images			Architecture	Performance Matrix	Explainable
	COVID-19	Normal	Others			
Loey et al. [28]	69	79	158	AlexNet, Google Net, Resnet18	Acc = 99%	NO
Civit-Masot et al. [25]	132	132	132	VGG16	avF1 = 0.85	NO
Altan et al. [24]	219	1341	1345	EfficientNetB	Acc = 99%	NO
Hemdan et al. [30]	25	50	-	VGG19, DenseNet	AvF1 = 0.90	NO
Narin et al. [26]	50	50	-	Inception v3, InceptionResNetv2, Resnet50	Acc = 98%	NO
Arias-Londono et al. [29]	7716	45,022	21,707	CovidNet	Acc = 91.53%	YES
Oh et al. [22]	180	191	131	Resnet18	Acc = 89%	YES
Khan et al. [21]	310	284	657	CoroNet	Acc = 89.5%	NO
Ozturk et al. [23]	127	500	600	DarkNet	Acc = 87%	NO
Zhang et al. [31]	100	1431	-	EficientNet	se = 96%, sp = 70%	NO
Asnaoui et al. [32]	48	11,203	1591	Inception Resnetv2	Acc = 92.2%	NO

**Table 2:** Performance measures for the three models considered in the article for two class classification.

Input Image Size	Models	Accuracy	Class	Precision (PPV)	Recall (Sensitivity)	F1 Score	AUC	Explainable AI
299 × 299	Xception	0.97	Normal	0.981	0.962	0.971	0.99	Y
			COVID	0.959	0.979	0.969	0.99	
224 × 224	VGG19	0.97	Normal	0.962	0.973	0.967	0.99	Y
			COVID	0.977	0.968	0.972	0.99	
224 × 224	Resnet50	0.925	Normal	0.958	0.892	0.923	0.97	Y
			COVID	0.896	0.959	0.926	0.97	
512 × 512	Xception	0.975	Normal	0.995	0.953	0.973	1	Y
			COVID	0.958	0.995	0.976	0.99	
	VGG19	0.975	Normal	0.961	0.99	0.975	0.99	Y
			COVID	0.99	0.961	0.975	0.99	
	Resnet50	0.933	Normal	0.983	0.881	0.93	0.98	Y
			COVID	0.89	0.985	0.935	0.98	

**Table 3:** Performance measures for the three models considered in the article for multi class classification.

Input Image Size	Models	Accuracy	Class	Precision (PPV)	Recall (Sensitivity)	F1 Score	AUC	Explainable AI
299 × 299	Xception	0.93	Normal	0.89	0.95	0.92	0.99	Y
			COVID	0.93	0.91	0.92	0.99	
			Lung Opacity	0.91	0.92	0.92	0.99	
			non-COVID viral pneumonia	0.98	0.94	0.96	0.99	
224 × 224	VGG19	0.92	Normal	0.87	0.97	0.92	0.99	Y
			COVID	0.94	0.88	0.91	0.99	
			Lung Opacity	0.87	0.92	0.89	0.99	
			non-COVID viral pneumonia	1	0.89	0.94	0.99	
224 × 224	Resnet50	0.75	Normal	0.8	0.9	0.85	0.96	Y
			COVID	0.65	0.78	0.7	0.89	
			Lung Opacity	0.73	0.49	0.59	0.87	
			non-COVID viral pneumonia	0.85	0.85	0.85	0.94	

## Data Availability

Data used to support the findings of the study are available in the manuscript.

## References

[1] WHO World Health Organization. Coronavirus Disease (COVID-19) Pandemic. https://www.who.int/europe/emergencies/situations/covid-19.

[2] WHO Novel Coronavirus (2019-Ncov)—World Health Organization. https://www.who.int/docs/default-source/coronaviruse/situation-reports/20200121-sitrep-1-2019-ncov.pdf?sfvrsn.

[3] World Health Organization Who Coronavirus (COVID-19) Dashboard. https://covid19.who.int/.

[4] Cui J., Li F., Shi Z.L. (2019). Origin and evolution of pathogenic coronaviruses. Nat. Rev. Microbiol..

[5] Centers for Disease Control and Prevention Coronavirus Disease 2019 (COVID-19). https://www.cdc.gov/coronavirus/2019-ncov/index.html.

[6] CDC Centers for Disease Control and Prevention. COVID-19 Vaccines for Specific Groups of People. Centers for Disease Control and Prevention. https://www.cdc.gov/coronavirus/2019-ncov/vaccines/recommendations/specific-groups.html.

[7] WHO World Health Organization. Tracking SARS-CoV-2 Variants. https://www.who.int/activities/tracking-SARS-CoV-2-variants.

[8] World Health Organization Criteria for Releasing COVID-19 Patients from Isolation. https://www.who.int/publications/i/item/criteria-for-releasing-covid-19-patients-from-isolation.

[9] Wang W., Xu Y., Gao R., Lu R., Han K., Wu G., Tan W. (2020). Detection of SARS-CoV-2 in Different Types of Clinical Specimens. JAMA-J. Am. Med. Assoc..

[10] Administrator J.H.C.H.S. Antigen and Molecular Tests for COVID-19. COVID-19 Testing Toolkit. https://www.centerforhealthsecurity.org/covid-19TestingToolkit/molecular-based-tests/current-molecular-and-antigen-tests.html.

[11] Sverzellati N., Ryerson C.J., Milanese G., Renzoni E.A., Volpi A., Spagnolo P., Bonella F., Comelli I., Affanni P., Veronesi L. (2021). Chest X-ray or CT for COVID-19 pneumonia? Comparative study in a simulated triage setting. Eur. Respir. J..

[12] RSNA Rural Areas Face Imaging Obstacles on the Road to Health Care Equity. https://www.rsna.org/news/2021/june/rural-radiology-equity.

[13] BC Emergency Medicine Network How Long Is Too Long for Emergent CT Imaging in Rural Communities?. https://www.bcemergencynetwork.ca/lounge/how-long-is-too-long-for-emergent-ct-imaging-in-rural-communities/.

[14] Bai H.X., Hsieh B., Xiong Z., Halsey K., Choi J.W., Tran T.M.L., Pan I., Shi L.B., Wang D.C., Mei J. (2020). Performance of Radiologists in Differentiating COVID-19 from Non-COVID-19 Viral Pneumonia at Chest CT. Radiology.

[15] Doctors, Nurses ‘Can’t Take Much More’ Amid COVID-19 Surge in Southern California. Physicians News. https://physiciansnews.com/2020/12/31/doctors-nurses-cant-take-much-more-amid-covid-19-surge-in-southern-california/.

[16] Harmon K. (2021). COVID-Overwhelmed Hospitals Strain Staff and Hope to Avoid Rationing Care. Sci. Am..

[17] Soda P., D’Amico N.C., Tessadori J., Valbusa G., Guarrasi V., Bortolotto C., Akbar M.U., Sicilia R., Cordelli E., Fazzini D. (2021). AIforCOVID: Predicting the clinical outcomes in patients with COVID-19 applying AI to chest-X-rays. An Italian multicentre study. Med. Image Anal..

[18] Santa Cruz B.G., Bossa M.N., Soelter J., Husch A.D. (2021). Public COVID-19 X-ray datasets and their impact on model bias-a systematic review of a significant problem. medRxiv.

[19] Liu J., Dong B., Wang S., Cui H., Fan D.P., Ma J., Chen G. (2021). COVID-19 lung infection segmentation with a novel two-stage cross-domain transfer learning framework. Med Image Anal..

[20] Wang L., Lin Z.Q., Wong A. (2020). COVID-Net: A tailored deep convolutional neural network design for detection of COVID-19 cases from chest X-ray images. Sci. Rep..

[21] Khan A.I., Shah J.L., Bhat M.M. (2020). CoroNet: A deep neural network for detection and diagnosis of COVID-19 from chest X-ray images. Comput. Methods Programs Biomed..

[22] Oh Y., Park S., Ye J.C. (2020). Deep Learning COVID-19 Features on CXR Using Limited Training Data Sets. IEEE Trans. Med. Imaging.

[23] Ozturk T., Talo M., Yildirim E.A., Baloglu U.B., Yildirim O., Rajendra Acharya U. (2020). Automated detection of COVID-19 cases using deep neural networks with X-ray images. Comput. Biol. Med..

[24] Altan A., Karasu S. (2020). Recognition of COVID-19 disease from X-ray images by hybrid model consisting of 2D curvelet transform, chaotic salp swarm algorithm and deep learning technique. Chaos Solitons Fractals.

[25] Civit-Masot J., Luna-Perejón F., Morales M.D., Civit A. (2020). Deep learning system for COVID-19 diagnosis aid using X-ray pulmonary images. Appl. Sci..

[26] Narin A., Kaya C., Pamuk Z. (2021). Automatic detection of coronavirus disease (COVID-19) using X-ray images and deep convolutional neural networks. Pattern Anal. Appl..

[27] Waheed A., Goyal M., Gupta D., Khanna A., Al-Turjman F., Pinheiro P.R. (2020). CovidGAN: Data Augmentation Using Auxiliary Classifier GAN for Improved COVID-19 Detection. IEEE Access.

[28] Loey M., Smarandache F., Khalifa N.E.M. (2020). Within the lack of chest COVID-19 X-ray dataset: A novel detection model based on GAN and deep transfer learning. Symmetry.

[29] Arias-Londono J.D., Gomez-Garcia J.A., Moro-Velazquez L., Godino-Llorente J.I. (2020). Artificial Intelligence applied to chest X-Ray images for the automatic detection of COVID-19. A thoughtful evaluation approach. IEEE Access.

[30] Hemdan E.E.D., Shouman M.A., Karar M.E. (2020). COVIDX-Net: A Framework of Deep Learning Classifiers to Diagnose COVID-19 in X-Ray Images. arXiv.

[31] Zhang J., Xie Y., Pang G., Liao Z., Verjans J., Li W., Sun Z., He J., Li Y., Shen C. (2021). Viral Pneumonia Screening on Chest X-Rays Using Confidence-Aware Anomaly Detection. IEEE Trans. Med. Imaging.

[32] El Asnaoui K., Chawki Y. (2020). Using X-ray images and deep learning for automated detection of coronavirus disease. J. Biomol. Struct. Dyn..

[33] Rahman T. COVID-19 Radiography Database. Kaggle. https://www.kaggle.com/datasets/tawsifurrahman/covid19-radiography-database.

[34] Chowdhury M.E., Rahman T., Khandakar A., Mazhar R., Kadir M.A., Mahbub Z.B., Islam K.R., Khan M.S., Iqbal A., Emadi N.A. (2020). Can AI Help in Screening Viral and COVID-19 Pneumonia?. IEEE Access.

[35] Medical Imaging Databank of the Valencia region BIMCV. BIMCV. https://bimcv.cipf.es/bimcv-projects/bimcv-covid19.

[36] Liob. Anonymized dataset of COVID-19 cases with a focus on radiological imaging. this includes images (x-ray/CT) with extensive metadata, such as admission-, ICU-, laboratory-, and patient master-data. GitHub. https://github.com/ml-workgroup/covid-19-image-repository.

[37] Haghanifar A., Majdabadi M.M., Choi Y., Deivalakshmi S., Ko S. (2020). COVID-cxnet: Detecting COVID-19 in frontal chest X-ray images using deep learning. Multimed. Tools Appl..

[38] RSNA Pneumonia Detection Challenge. Kaggle. https://www.kaggle.com/c/rsna-pneumonia-detection-challenge.

[39] Mooney P. Chest X-ray images (pneumonia). Kaggle. https://www.kaggle.com/datasets/paultimothymooney/chest-xray-pneumonia.

[40] Haghanifar A., Majdabadi M.M., Choi Y., Deivalakshmi S., Ko S. (2020). COVID-CXNet: Detecting COVID-19 in Frontal Chest X-ray Images using Deep Learning. arXiv.

[41] Redazione. COVID-19 Database. SIRM. https://sirm.org/category/covid-19/.

[42] Cohen J.P., Morrison P., Dao L. (2020). COVID-19 Image Data Collection. arXiv.

[43] Goodman L.R. (2014). Felson’s Principles of Chest Roentgenology, a Programmed Text.

[44] Simonyan K., Zisserman A. (2014). Very deep convolutional networks for large-scale image recognition. arXiv.

[45] He K., Zhang X., Ren S., Sun J. (2016). Deep residual learning for image recognition. Proc. IEEE Comput. Soc. Conf. Comput. Vis. Pattern Recognit..

[46] Deng J., Dong W., Socher R., Li L.J., Li K., Fei-Fei L. Imagenet: A large-scale hierarchical image database. Proceedings of the 2009 IEEE Conference on Computer Vision and Pattern Recognition.

[47] Chollet F. Xception: Deep learning with depthwise separable convolutions. Proceedings of the IEEE Conference on Computer Vision and Pattern Recognition.

[48] van Rijsbergen C.J. (1971). An algorithm for information structuring and retrieval. Comput. J..

[49] Selvaraju R.R., Cogswell M., Das A., Vedantam R., Parikh D., Batra D. (2020). Grad-CAM: Visual Explanations from Deep Networks via Gradient-Based Localization. Int. J. Comput. Vis..

